# Functional Activity of the Complement System in Hospitalized COVID-19 Patients: A Prospective Cohort Study

**DOI:** 10.3389/fimmu.2021.765330

**Published:** 2021-10-28

**Authors:** Panteleimon Charitos, Ingmar A. F. M. Heijnen, Adrian Egli, Stefano Bassetti, Marten Trendelenburg, Michael Osthoff

**Affiliations:** ^1^ Division of Internal Medicine, University Hospital Basel, Basel, Switzerland; ^2^ Division of Medical Immunology, Laboratory Medicine, University Hospital Basel, Basel, Switzerland; ^3^ Clinical Bacteriology and Mycology, Laboratory Medicine, University Hospital Basel, Basel, Switzerland; ^4^ Applied Microbiology Research, Department of Biomedicine, University of Basel, Basel, Switzerland; ^5^ Department of Biomedicine, University of Basel, Basel, Switzerland; ^6^ Department of Clinical Research, University of Basel, Basel, Switzerland

**Keywords:** COVID-19, C1 esterase inhibitor, SARS-CoV-2, inflammation, complement system, mannose-binding lectin, ficolin-3

## Abstract

**Aims:**

Although the exact factors promoting disease progression in COVID-19 are not fully elucidated, unregulated activation of the complement system (CS) seems to play a crucial role in the pathogenesis of acute lung injury (ALI) induced by SARS-CoV-2. In particular, the lectin pathway (LP) has been implicated in previous autopsy studies. The primary purpose of our study is to investigate the role of the CS in hospitalized COVID-19 patients with varying degrees of disease severity.

**Methods:**

In a single-center prospective observational study, 154 hospitalized patients with PCR-confirmed SARS-CoV-2 infection were included. Serum samples on admission to the COVID-19 ward were collected for analysis of CS pathway activities and concentrations of LP proteins [mannose-binding lectin (MBL) and ficolin-3 (FCN-3)] & C1 esterase inhibitor (C1IHN). The primary outcome was mechanical ventilation or in-hospital death.

**Results:**

The patients were predominately male and had multiple comorbidities. ICU admission was required in 16% of the patients and death (3%) or mechanical ventilation occurred in 23 patients (15%). There was no significant difference in LP activity, MBL and FCN-3 concentrations according to different peak disease severities. The median alternative pathway (AP) activity was significantly lower (65%, IQR 50-94) in patients with death/invasive ventilation compared to patients without (87%, IQR 68-102, p=0.026). An optimal threshold of <65.5% for AP activity was derived from a ROC curve resulting in increased odds for death or mechanical ventilation (OR 4,93; 95% CI 1.70-14.33, p=0.003) even after adjustment for confounding factors. Classical pathway (CP) activity was slightly lower in patients with more severe disease (median 101% for death/mechanical ventilation vs 109%, p=0.014). C1INH concentration correlated positively with length of stay, inflammatory markers and disease severity on admission but not during follow-up.

**Conclusion:**

Our results point to an overactivated AP in critically ill COVID-19 patients *in vivo* leading to complement consumption and consequently to a significantly reduced AP activity *in vitro.* The LP does not seem to play a role in the progression to severe COVID-19. Apart from its acute phase reaction the significance of C1INH in COVID-19 requires further studies.

## Introduction

In December 2019, a novel coronavirus was identified as the cause of a cluster of pneumonia cases in Wuhan, a city in the Hubei Province of China. It rapidly spread, resulting in an epidemic throughout China, followed by an unprecedented worldwide pandemic with more than 202 million identified cases and more than 4 million deaths (1). In February 2020, the World Health Organization designated the disease COVID-19, which stands for coronavirus disease 2019. The virus that causes COVID-19 was designated severe acute respiratory syndrome coronavirus 2 (SARS-CoV-2). The clinical spectrum of COVID-19 ranges from asymptomatic carriers to respiratory failure requiring respiratory support in the intensive care unit (ICU). In the latter setting, a dysregulated immune response characterized by a decrease in suppressor T cell counts, excessive release of pro-inflammatory cytokines, and activation of several inflammatory cascades including the contact activation, coagulation and complement cascade, contributes to the observed organ dysfunction (2–4).

The complement system is an integral part of the innate immune system and acts as a first line of defence by inducing an inflammatory response after opsonisation of pathogens and dying cells (5, 6). The complement cascade is initiated by at least three pathways, i.e. the classical, the lectin, and the alternative pathway. The lectin pathway (LP) of complement is activated after binding of its pattern-recognition receptors (PRR) including mannose-binding lectin (MBL) and the ficolins to carbohydrate patterns, acetyl groups or immunoglobulin M with subsequent activation of MBL-associated serine protease (MASP)-1 and -2 and assembly of the C3 convertase (7). Inter-individual serum concentrations of LP PRR vary to a considerable degree with the greatest differences observed in MBL levels (from undetectable to about 10 µg/mL) (8, 9).

The complement system and particularly the LP has been found to interact with and be involved in the clearance of a number of viruses (10–14). Importantly, its contribution to the risk and severity of SARS-CoV, that emerged in 2002 to 2003 and led to a global outbreak of SARS, has been evaluated in detail previously. Binding of MBL to SARS-CoV was demonstrated, which consequently interfered with efficient viral entry (15, 16). More importantly, several case-control studies have documented an increased susceptibility to SARS-CoV infection in patients with low MBL concentrations or certain low-producing genotypes (16–18) but not for the presence of MASP-2 polymorphisms (19). In COVID-19, convincing data has emerged showing that complement system activation mediates thrombo-inflammation and may contribute to respiratory failure and mortality (20, 21). Regarding the lectin pathway, MASP-2 was found to interact with SARS-CoV-2 leading to uncontrolled activation of the complement cascade (22). In line, pulmonary vascular MASP-2 deposits were demonstrated in an autopsy study of critically ill COVID-19 patients, and MBL concentrations were higher in patients with thromboembolic events (21, 23). However, data on the role of the LP are controversial. In a recent study, *MBL2* variants associated with lower MBL levels were more frequently encountered in COVID-19 compared to control patients and were associated with a more severe disease suggesting a protective role of MBL (24).

Ficolin-3 (FCN3) is the most abundant of the three ficolins in serum, and the only one that is expressed in the lungs even exceeding its expression in the liver (25). Although data on SARS-CoV-2 are lacking, interactions with seasonal influenza viruses have been described (26, 27). In a small study, increased FCN3 concentrations were observed in severe COVID-19 in Asian patients with significant renal disease (28). Lastly, C1 esterase inhibitor (C1INH) is a serine-protease inhibitor of manifold targets including the classical pathway (CP) and LP of the complement system (29). In line with its role as an acute phase protein, elevated serum concentrations have been documented in severe COVID-19 (30, 31), whereas its expression in bronchoalveolar lavage samples was significantly decreased (32). Interestingly, C1INH was able to block MASP-2 mediated overactivation of the complement system and lung injury induced by several pathogenic coronaviruses (22).

With regards to COVID-19, it remains to be elucidated, which complement pathway crucially contributes to complement activation and its clinical consequences. Previous studies have pointed to the LP and alternative pathway (AP) (31, 33–35).

Given the paucity of data regarding the lectin pathway of complement in SARS-CoV-2 infection, we aimed to investigate serum concentrations of two important PRR of the LP and of its predominant inhibitor (i.e. C1INH) as well as to clarify the role of complement pathway activities in a well-characterized cohort of COVID-19 patients with respect to severity and outcome.

## Materials and Methods

### Ethics Statement

The study protocol was approved by the Ethics Committee of Northwest and Central Switzerland (EKNZ 2020-00769) with a waiver for informed consent. The study was performed in accordance with the latest version of the declaration of Helsinki and the guidelines for good clinical practice.

### Patient Inclusion and Sample Collection

This prospective observational cohort study was performed at a single tertiary care center in Switzerland. Consecutive patients with a SARS-CoV-2 infection confirmed by polymerase chain reaction (PCR) from a nasopharyngeal swab and admitted to the hospital between March and May 2020 were included. Serum samples were collected on admission to the COVID-19 ward during routine blood sampling, transferred to the laboratory immediately after sampling, centrifuged, aliquoted and stored at −80°C until measurement of the complement pathway activities and concentrations of MBL, FCN-3 and C1INH. According to the institutional protocol at that time most patients received lopinavir/ritonavir and hydroxychloroquine treatment, but not corticosteroids. In addition, patients who deteriorated with a C-reactive protein (CRP) above 70 mg/L and/or progressive lung involvement on repeat chest computed tomography (CT) scan were treated with tocilizumab (8 mg/kg body weight up to 800 mg with a repeat dose after 24 to 48 hours if required) on a compassionate use basis.

Patients were excluded if they had refused the general research consent of our institution, if a serum sample was not available on admission or if patients were transferred to another institution within 72 hours (lost to follow-up).

### Laboratory Assessment

Complement pathway activities (WIESLAB^®^ Complement system Screen kit, Svar Life Science AB, Sweden) and C1INH antigen concentration (Siemens, Marburg, Germany) were determined on semi-automated platforms in the Clinical Laboratory of the University Hospital according to the manufacturer’s instruction and standard operating procedures. Low CP and AP activities are usually caused by *in-vivo* activated pathways with consumption of complement components, whereas low LP activity is usually due to low MBL concentrations.

For FCN-3 a research-use-only enzyme-linked immunosorbent assay (ELISA) kit was used according to the manufacturer’s instruction (Hycult Biotech, Uden, the Netherland). MBL concentration was quantified using an in-house ELISA with mannan coating as previously described (36). A biotinylated mouse anti-human MBL antibody (HYB131-01B, Bioporto Diagnostics, Denmark) was used for detection and a pooled human serum with known MBL concentration (BioPorto Diagnostics, Denmar) was used to generate a standard curve.

### Primary and Secondary Outcomes and Data Extraction

The primary outcome of this study was a composite clinical outcome combining in-hospital all-cause mortality and need of intubation and mechanical ventilation, corresponding to a score of 6-8 on the WHO ordinal scale for clinical improvement (37). Secondary outcomes were length of stay (LOS) until discharge to home or a non-acute care (rehabilitation) center or death occurred and correlation with inflammatory markers.

Clinical data and laboratory results were collected from the electronic health records and recorded in an electronic database (Research Electronic Data Capture). Patients were followed up for the primary and secondary clinical outcome until death or discharge occurred.

Semi-automatic quantification of affected lung tissue on a CT scan was performed using software for lung density analysis in Chest CT [CT Pulmo 3D included in Syngo.Via VB30A, Siemens Healthineers, Forchheim, Germany; method similar as described (38)]. After semi-automatic segmentation of the lungs a threshold analysis of Hounsfield units (HU) was pursued, where pulmonary involvement was defined as the percentage of lung parenchyma with a CT-density between -600 and 0 HU.

### Statistical Analysis

Statistical analysis was performed using SPSS software, version 25.0 (IBM, USA), and GraphPad Prism 7 software was used for visualization (GraphPadSoftwares Inc., La Jolla, Ca, USA). Most of the variables showed skewed distributions and for this reason data are represented as medians with interquartile range (IQR), if not mentioned otherwise. Data from normally distributed variables are presented as means with standard deviation (SD). Nominal data are presented as frequencies (%). Differences between patient groups were assessed using the Mann-Whitney U test for continuous variables, and the chi-square or Fisher’s exact test for nominal variables. Correlations between complement factors and the secondary clinical outcome (LOS) or inflammatory markers were assessed using Spearman rank correlation tests. Multivariable logistic regression models were performed to analyze associations between complement parameters and the composite outcome of mechanical ventilation and/or in-hospital death. Results are presented as odds ratios (OR) with corresponding 95% confidence intervals (CI). Variable selection was based on biologic plausibility and/or demonstrated associations. Differences between groups or correlations were considered statistically significant at the level of p < 0.05 (2-tailed).

## Results

### Patient Characteristics

Overall, 154 of 189 patients with PCR-confirmed SARS-CoV-2 infection and hospitalized during the first wave were included in this study. Patients were predominately male (94/154, 61%) with a median age of 62 years (IQR 49-73). Frequent comorbidities included arterial hypertension, obesity (body mass index (BMI) ≥30 kg/m^2^], and cardiovascular disease (**Table 1**). The median duration of symptoms before admission was 7 days [IQR 3-11). 57 patients (37%) had a SOFA score of at least 2 points on admission, indicating severe disease. Median peak viral load in nasopharyngeal swab samples and median peak affected lung volume on computed tomography scan of the chest were 118’900 copies/ml (IQR 9’700-1’705’825) and 14% (IQR 6.6-25) of the total lung volume, respectively. Further demographic and baseline characteristics are reported in **Table 1**.

**Table 1:** Demographic and baseline characteristics, therapeutic management and outcomes of the entire patient cohort and according to the composite outcome of mechanical ventilation and/or in-hospital death.

Variables	Total n=154	Patients without the composite outcome n=131	Patients with the composite outcome n=23	p-values
Demographics				
Male sex, n (%)	94 (61)	77 (59)	17 (74)	0.170
Age on admission in years, mean (SD)	61 (16)	60 (16)	65 (14)	0.205
Body mass index (BMI) in kg/m^2,^ median (IQR)	27 (24-32)	27 (24-31)	30 (26-34)	0.093
Comorbidities				
Arterial hypertension, n (%)	81 (53)	64 (49)	17 (74)	**0.026**
Obesity (BMI>=30 kg/m^2^), n (%)	52 (34)	40 (31)	12 (52)	**0.043**
Diabetes mellitus, n (%)	32 (21)	26 (20)	6 (26)	0.496
Chronic lung disease, n (%)	31 (20)	24 (18)	7 (30)	0.181
Cardiovascular disease, n (%)	46 (30)	36 (27)	10 (43)	0.122
Chronic renal failure, n (%)	25 (16)	19 (15)	6 (26)	0.170
Solid or haematological cancer, n (%)	21 (14)	17 (13)	4 (17)	
Immunosuppression, n (%)	21 (14)	17 (13)	4 (17)	0.569
Charlson comorbidity index, median (IQR)	3 (1-5)	2 (1-5)	4 (2-7)	0.109
Clinical characteristics				
Symptom duration before admission in days, median (IQR)	7 (3-11)	7 (3-11)	8 (6-12)	0.449
SOFA score on admission, median (IQR)	1 (0-2)	1 (0-2)	2 (1-3)	**0.000**
NEWS2 score on admission, median (IQR)	3 (2-5)	3 (2-5)	4.5 (2.5-7.5)	**0.028**
Oxygen saturation on admission, median (IQR)	95 (93-97)	96 (94-97)	93 (90-96)	**0.001**
Presenting symptoms				
Cough, n (%)	101 (66)	86 (66)	15 (65)	0.968
Fever, n (%)	91 (59)	74 (56)	17 (74)	0.117
Shortness of breath, n (%)	54 (35)	45 (34)	9 (39)	0.658
Routine laboratory findings (with normal ranges) on admission, median (IQR)				
Leukocytes x10^9^/l (3.5-10.0)	6.0 (4.2-8.2)	6.0 (4.1-8.2)	5.9 (4.8-8.1)	0.94
Lymphocytes x10^9^/l (0.9-3.3)	1.0 (0.6-1.3)	1.0 (0.7-1.4)	0.7 (0.5-1.1)	**0.014**
Platelets x10^9^/l (150-450)	206 (146-242)	209 (150-275)	171 (131-220)	**0.032**
C-reactive protein in mg/l (<10.0)	39.5 (16.4-76.2)	38 (14.5-72.1)	72.2 (33.5-112.6)	**0.016**
Interleukin-6 in ng/l (<7.0)	80.8 (36.1-107)	57.4 (24.8-87.3)	105.0 (56.9-210.5)	**0.003**
Ferritin in μg/l (10-200)	586 (288-1234)	555 (279-1202)	865 (406-2243)	0.146
D-dimer in μg/l (0.19-0.50)	0.72 (0.42-1.58)	0.73 (0.40-1.68)	0.66 (0.47-1.27)	1.0
LDH in IU/l (135-225)	269 (222-360)	265 (218-352)	340 (232-455)	0.085
Peak viral load in nasopharyngeal swab in copies/ml	118`900 (9`700-1`705`825)	78`900 (7`900-1`425`600)	692`400 (115`100-3`095`900)	**0.02**
Peak affected lung volume as percentage (%) on computed tomography scan of the chest, median (IQR)	14.0 (6.6-25.0)	12.0 (6.0-20.1)	29.0 (24.3-36.3)	**<0.001**
Treatment				
Lopinavir/ritonavir, n (%)	100 (65)	81 (62)	19 (83)	0.054
Hydroxychloroquine, n (%)	125 (81)	103 (79)	22 (96)	0.054
Remdesivir, n (%)	7 (5)	0 (0)	7 (30)	**<0.001**
Tocilizumab, n (%)	42 (27)	25 (19)	17 (74)	**<0.001**
Antibiotics, n (%)	67 (44)	46 (35)	21 (91)	**<0.001**
Outcomes				
In-hospital mortality, n (%)	4 (3)	0 (0)	4 (17)	**<0.001**
ICU admission, n (%)	25 (16)	6 (5)	19 (83)	**<0.001**
[with median (IQR) LOS in ICU in days]	[8 (3-13)]	[1 (1-3)]	[10 (6-13)]	
LOS in days, median (IQR)	8 (5-11)	6 (4-10)	20 (12-28)	**<0.001**

BMI, body mass index; ICU, intensive-care unit; IQR, interquartile range; LDH, lactate dehydrogenase; LOS, length of stay; NEWS2, National Early Warning Score 2; SOFA, sepsis-related organ failure assessment score; SD, standard deviation.

Statistically significant results (*p* < 0.05) are marked in boldface font.

In total, 25 patients (16%) required transfer to the intensive care unit (ICU) during the disease course, 10 (8%) of whom were transferred less than 24 hours after their initial admission to the COVID-19 ward. Death (4/154, 3%) and/or mechanical ventilation occurred in 23 patients (15%). These patients suffered more frequently from arterial hypertension or obesity, presented with more advanced disease, and were more likely to receive antibiotic, antiviral and anti-inflammatory therapies (**Table 1**).

### Association With Disease Severity

**Figure 1:**
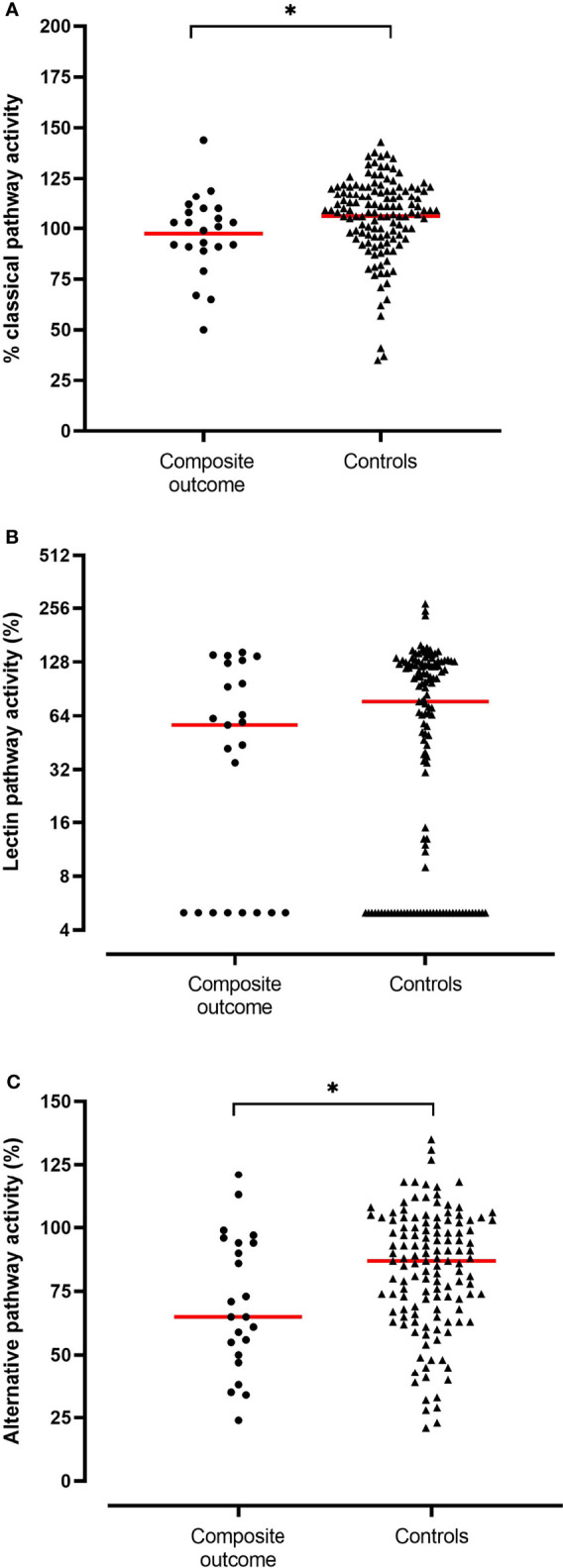
Activity levels (in %) of the classical **(A)**, lectin **(B)** and alternative pathway **(C)** in patients who required mechanical ventilation or died during the hospitalization (n = 23, composite outcome) compared to patients who survived without requiring mechanical ventilation (n = 131, controls). Medians are depicted (red line). Significant differences (p<0.05) are marked with an asterisk.

Neither FCN-3, MBL, C1INH serum concentrations nor LP activity on admission were associated with the occurrence of the composite outcome of mechanical ventilation or in-hospital death (**Table 2**). Furthermore, moderate and severe MBL deficiencies, defined as serum concentration below 500 ng/ml and below 100 ng/ml, respectively, were not associated with the clinical outcome. In contrast, CP activity was slightly lower and AP activity significantly lower in patients who required mechanical ventilation and/or died [CP activity, median (IQR) 101% (91-110) vs. 109% (97-119), p=0.014; AP activity, median (IQR) 65% (50-94) vs. 87% (68-102), p=0.026] (**Figure 1**) suggesting complement consumption due to *in-vivo* activation *via* the AP and to a lesser degree *via* the CP of complement. Due to the larger median difference, we explored receiver-operator characteristics analysis of the AP activity to evaluate if a dichotomous cut-off might differentiate between patients with and without the composite outcome (**Figure S1**). An optimal cut-off of 65.5% for the AP activity was derived from this analysis (AUC 0.656, p=0.026) and was tested in the multivariable analysis. Patients with an AP activity of less than 65.5% on admission had higher odds for in-hospital death or mechanical ventilation (OR 4.93 (IQR 1.70-14.33), p = 0.003) after adjustment for comorbidities and disease severity on admission (**Table 3**).

**Table 2:** Complement parameters in the entire cohort and according to the composite outcome of mechanical ventilation and/or in-hospital death.

Complement parameter	Total n=154	Patients without the composite outcome n=131	Patients with the composite outcome n=23	p-values
Median (IQR) or n (%)				
Lectin pathway activity, %	69 (5-126)	77 (5-126)	57 (5-112)	0.392
Classical pathway activity, %	108 (95-119)	109 (97-119)	101 (91-110)	**0.014**
Alternative pathway activity, %	86 (65-100)	87 (68-102)	65 (50-94)	**0.026**
MBL in ng/ml	1`913 (261-4419)	1`858 (305-4`320)	2`280 (36-5`177)	0.763
MBL < 500 ng/ml	44 (28.6)	37 (28.2)	7 (30.4)	0.805
MBL < 100 ng/ml	32 (20.8)	25 (19.1)	7 (30.4)	0.260
FCN-3 in ng/ml	40`728 (28`036-50`971)	41`063 (30`121-51`080)	34’681 (23`725-48`444)	0.341
C1INH in g/l	0.47 (0.40-0.54)	0.46 (0.40-0.54)	0.50 (0.41-0.57)	0.444

C1INH, C1 esterase inhibitor; FCN-3, ficolin-3; MBL, mannose-binding lectin.

Statistically significant results (*p* < 0.05) are marked in boldface font.

**Table 3:** Predictors of mechanical ventilation or in-hospital death in the multivariable analysis.

Variable	Multivariable OR (95% CI)	P-value
Alternative pathway activity <65.5%	4.93 (1.70-14.33)	**0.003**
Obesity	2.96 (1.02-8.58)	**0.046**
Arterial Hypertension	2.15 (0.65-7.10)	0.211
SOFA score on admission (per 1 point increase)	1.59 (1.02-2.16)	**0.038**
CRP on admission (per 1 mg/L increase)	1.01 (0.99-1.01)	0.167

CI, confidence interval; CRP, C-reactive protein; OR, odds ratio; SOFA, sepsis-related organ failure assessment score.Statistically significant results (p < 0.05) are marked in boldface font.

Regarding the secondary outcomes, only C1INH and the AP activity showed a weak correlation with disease severity on admission as assessed by the SOFA score. Only C1INH correlated weakly with length of stay (r = 0.266, p = 0.002; **Table 4**). In line, an AP activity below 65.5% was more frequently encountered in patients with a SOFA score of at least 2 on admission (38.6% vs. 20.6%, p=0.016).

**Table 4:** Correlation of complement pathway activities and protein concentrations and C1INH with length of stay, disease severity on admission and inflammatory markers on admission.

Complement variables	LOS,	SOFA score	CRP	Ferritin	IL-6	D-Dimer	Lymphocyte count	LDH	Peak SARS-CoV-2 viral load in nasopharyngeal swab
r (p-value)*									
Lectin pathway activity	0.008 (0.926)	-0.013 (0.870)	**0.172 (0.041)**	**0.269 (0.006)**	-0.184 (0.237)	0.041 (0.697)	-0.001 (0.988)	0.060 (0.461)	-0.099
									(0.314)
Classical pathway activity	-0.039 (0.629)	-0.016 (0.847)	**0.172 (0.040)**	**0.229 (0.020)**	**-0.316 (0.039)**	0.017 (0.874)	0.044 (0.607)	0.106 (0.194)	-0.033
									(0.736)
Alternative pathway activity	-0.104 (0.200)	**-0.163 (0.044)**	-0.048 (0.570)	-0.007 (0.945)	**-0.338 (0.027)**	**-0.207 (0.046)**	**0.277 (0.001)**	-0.153 (0.061)	-0.070
									(0.478)
MBL	0.053 (0.524)	0.041 (0.624)	**0.234 (0.006)**	**0.364 (0.000)**	-0.079 (0.628)	0.091 (0.392)	-0.035 (0.691)	0.134 (0.107)	-0.097
									(0.330)
FCN-3	-0.103 (0.212)	-0.123 (0.135)	**0.170 (0.046)**	0.103 (0.307)	-0.103 (0.517)	-0.200 (0.054)	-0.083 (0.336)	0.152 (0.066)	-0.076
									(0.441)
C1INH	**0.266 (0.002)**	**0.224 (0.009)**	**0.455 (0.000)**	**0.477 (0.000)**	0.019 (0.914)	0.103 (0.358)	**-0.240 (0.008)**	**0.491 (0.000)**	0.048
									(0.647)

*Spearman correlation coefficients and p-values are presented.

C1INH, C1 esterase inhibitor; CRP, C-reactive protein; FCN-3, ficolin-3; IL-6, interleukin-6; LOS, length of stay; MBL, mannose-binding lectin; r, Spearman correlation coefficient; p, p-value; SARS-CoV-2, severe acute respiratory syndrome coronavirus type 2; SOFA, sepsis-related organ failure assessment score.

Statistically significant results (*p* < 0.05) are marked in boldface font.

### Correlation With Inflammation

Admission CP, LP activity and MBL concentrations correlated only weakly with CRP and ferritin sampled at the same time point, but not with D-Dimer or lymphocyte count (**Table 4**). In line, MBL deficiency < 500 ng/ml was only associated with a lower ferritin concentration on admission [median 433 µg/l (IQR 238-738) vs. 844 µg/l (IQR 335-1392) in patients without MBL deficiency < 500 ng/ml, p=0.008]. For FCN-3, correlations with inflammatory markers were essentially absent, whereas C1INH – the natural inhibitor of the LP and CP – correlated positively with several inflammatory markers on admission, in particular CRP (r = 0.455, p < 0.001) and ferritin (r = 0.477, p < 0.001 (**Table 4**). In addition, C1INH correlated positively with peak CRP (r = 0.469, p<0.001), peak ferritin and lactate dehydrogenase concentrations (r = 0.546, p<0.001 and r = 0.545, p<0.001, respectively), and peak affected lung volume on CT scan of the chest (r = 0.381, p<0.001). Interestingly, a higher C1INH concentration on admission was associated with the subsequent administration of tocilizumab [median 0.51 (IQR 0.46-0.57) g/L vs. 0.45 (IQR 0.39-0.53) g/L in patients not receiving tocilizumab, p=0.003].

## Discussion

The complement system has been implicated in the pathogenesis and severity of COVID-19 in several studies (20, 21, 35). The present study is the largest study to assess serum MBL, ficolin-3 and C1INH concentrations as well as CP, AP and LP activity in a well-characterized cohort of COVID-19 patients. In addition, it extends previous data on the role of the AP in COVID-19 by presenting data on the association of AP activity measured on admission with a composite outcome of mechanical ventilation and/or in-hospital death.

Neither serum concentrations of MBL and FCN-3 nor LP activity on admission were associated with mechanical ventilation and/or in-hospital death, and only a very weak correlation with markers of inflammation was observed. This is surprising given the proposed involvement of MBL and the LP in the pathogenesis of SARS-CoV-2 infection. MBL and other pattern-recognition receptors of the LP bind directly to SARS-CoV-2 nucleocapsid and spike proteins, and LP activation was demonstrated upon binding (39). In addition, complement mediated injury has been documented in the lungs and skin of patients with severe COVID-19 with significant deposition of MASP-2, MBL and even ficolin-3 (21, 40), and MASP-2 deficient mice were protected from severe disease (22). Lastly, Shen B et al. documented a significant upregulation of mannose in sera of severe COVID-19 patients, which may lead to complement activation upon binding of MBL to mannose (41). Our results are in line with observations from smaller cohorts that have not found a an association of MBL or FCN-3 serum concentrations (20, 23, 28) or LP activity (34) with outcome or severity in COVID-19. In contrast, MBL (elevated proteomic signature) was found to be associated with 28-day mortality in a proteomic analysis (31). However, this study only included sera from 62 ICU patients, and the predictive performance of the MBL proteomic signature could not be tested in the much larger validation cohort (31). Of note, MBL genotypes associated with lower protein concentrations (i.e. the opposite as observed in the proteomic study) were more frequently encountered in patients with severe disease and need for ICU support in a study from Turkey (24). Lastly, a small study of 65 ICU patients reported no association of MBL with survival or need of mechanical ventilation similar to our cohort but higher MBL levels in patients with thromboembolic events, which correlated with D-dimer levels (23). Due to a very low number of thromboembolic events we were not able to confirm or refute this finding in the present study, but MBL concentrations or activity did not correlate with D-dimer levels in our cohort, even when limiting the analysis to ICU patients (data not shown). In summary, current human studies measuring LP proteins or activity have not been able to support the current concept of a core role of the LP in the overactivation of the complement system and the pathogenesis of COVID-19.

In contrast to the LP, low AP activity measured on admission to the COVID-19 and suggesting *in-vivo* overactivation *via* the AP emerged as a significant predictor of mechanical ventilation or in-hospital death in the present analysis. Patients with AP activity below the cut-off derived from the ROC analysis were almost five times more likely to develop acute lung injury requiring mechanical ventilation and/or to die compared to patients with AP activity above this cut-off. In addition, a lower AP activity also correlated with a higher disease severity on admission as assessed by the SOFA score. Our study cohort is the largest to date assessing AP activity and provides independent evidence regarding the predictive value of AP activity when assessed early during admission for COVID-19. Sinkovits G et al. have previously assessed AP activity in a cohort of 102 COVID-19 patients, too (34). However, AP activity was not uniformly measured on admission (but up to 63 days later; one third of patients were already in the ICU during sampling). They report decreased AP activity in critically ill patients at the time of sampling compared to non-ICU patients and outpatients. In addition, AP activity was significantly lower in deceased patients compared to ICU or non-ICU patients. Our results extend their findings by demonstrating that a decreased AP activity on admission is associated with a poor outcome in COVID-19. AP activity was also measured in a subgroup of patients (n=38) in the study by Ma L et al. but was found not to be significantly different when comparing ICU vs. non-ICU patients, a result that is probably influenced by the small number of patients analyzed (33). The measurement of AP activity provides a global assessment and characterization of several proteins that regulate AP activity and determine total AP function. As such, our analyses suggest that the occurrence of an overactivated AP and consumption of its associated complement proteins already on admission to the COVID-19 ward may be associated with a worse prognosis. In line, several studies have documented higher concentrations of complement activation products upstream of the terminal complement pathway (such as C3a) being associated with ICU admission, death and thromboembolic events (23, 33, 34). Both, Ma L et al. and Sinkovits G et al. reported that markers of AP activation and consumption may identify SARS-CoV-2 infected patients with a poor prognosis (33, 34). For example, the ratios of C3a/C3 and iC3b/C3 were associated with mortality and ICU admission, respectively (33, 34), and factor D strongly correlated with markers of endothelial cell activation and coagulation (33). Interestingly, SARS-CoV-2 spike protein has been found to activate the alternative pathway directly (42), which was blocked by factor D inhibition. Apart from the AP, the CP seems to be overactivated as a result of SARS-CoV-2 infection in our cohort. These data support a role of the C3 and the AP axis in complement activation in severe COVID-19 either as a consequence of direct activation by SARS-CoV-2 or as an amplifier secondary to CP (and to a lesser degree LP) activation.

We observed markedly elevated C1INH concentrations already on admission (median 0.47 g/L; upper limit of normal of 0.39 g/l at our institution). This should be viewed as biological feedback mechanisms in response to activation of several plasmatic cascades including the complement system in an attempt to inhibit or control inappropriate activation (43), which has also been documented in sepsis patients (44). C1INH was the most significantly upregulated protein in COVID-19 patients compared to controls with COVID-19 like symptoms in a preprint study (45). Similarly, a significant increase in C1INH protein signature was documented in severe compared to non-severe COVID-19 patients (41). In line, C1INH levels correlated positively not only with inflammatory markers on admission but also with their peak values in the present study. Moreover, C1INH levels on admission provided information on peak lung involvement as assessed on CT scans of the chest and on subsequent escalation of treatment (tocilizumab in our center). However, C1INH was not associated with the composite outcome in agreement with a previous smaller study (46). Given that C1INH is only a very weak regulator of the alternative pathway the lack of its association may support the observed significant association of AP activity with the outcome in our study. As even elevated C1INH concentrations may not be sufficient to inhibit or modulate all potential downstream effectors within the complement and contact activation cascade (47) and due to the detection of an increased amount of modified (cleaved) inactive C1INH in patients with severe sepsis (48) and a decreased expression of C1INH in the lungs of COVID-19 patients (32), results from trials evaluating additional C1INH supplementation treatment (49, 50) will be informative if C1INH may influence COVID-19 severity and outcome independent of AP activation (e.g. by inactivating the contact activation system or MASP-2).

Our study did not include a control population of SARS-CoV-2 negative patients with similar disease severity (e.g. influenza). This is an important limitation, as AP activation and consumption and its association with severe SARS-CoV-2 infection and detrimental outcomes may not be specific for COVID-19. In fact, Bain W et al. demonstrated that decreased AP activity was associated with an increased 30-day and one-year mortality in critically ill patients with acute lung injury as a consequence of infectious and non-infectious etiologies (51). Interestingly, a “hyperinflammatory” subphenotype was more frequently observed in patients with decreased AP activity as were bloodstream infections. This may point towards a detrimental effect of an overactive AP leading to a diminished AP function, in particular if sustained over time. In line, Bibert S et al. observed a similar pattern of increased expression of complement component-encoding genes in COVID-19 and influenza compared to healthy controls (52) with the exception of C3 that was over-expressed in early COVID-19 compared to influenza patients.

Our study has several additional limitations including the lack of data on other important proteins of the LP (other ficolins, collectin liver 1, MASP-2 and MASP-3) and AP and the single-center design. Importantly, none of our patients was treated with corticosteroids, which were not yet standard of care at the time of inclusion of patients. On the other hand, a significant number of patients was already treated with tocilizumab, a therapy that has shown to improve outcome in moderate to severe COVID-19 (53, 54). Consequently, outcome results generated in this study may even be valid in the current area of COVID-19 treatment. In-hospital mortality was lower than reported for our center during the first wave [e.g. 3% vs. 9.5% for in-hospital mortality (55)] and much lower compared to reported rates in the literature, which may have influenced our results. In particular, a serum sample may have not been available on admission of patients that required immediate transfer to the ICU from the emergency department (and subsequently died). Adjustment for confounders in the multivariable analysis of the composite outcome was limited. The significance of our results is limited by the small sample size of the analyzed cohort and the multiple comparisons investigated. Significant differences as described might be a chance result in the setting of multiple statistical analyses. Vice versa, it is possible that small differences in the composite outcome according to lectin pathway protein or C1INH concentrations may only be detectable in a larger patient cohort. Lastly, protein concentrations were only determined on admission but not during the disease course or at admission to the ICU.

## Conclusion

Our results point to an overactivated AP in critically ill COVID-19 patients *in-vivo* leading to complement consumption and consequently to a significantly reduced AP activity *in-vitro.* The LP does not seem to play a role in the progression to severe COVID-19. Apart from its acute phase reaction the significance of C1INH in COVID-19 requires further studies.

## Data Availability Statement

The original contributions presented in the study are included in the article/**Supplementary Material**. Further inquiries can be directed to the corresponding author.

## Ethics Statement

The studies involving human participants were reviewed and approved by Ethics Committee of Northwest and Central Switzerland. Written informed consent for participation was not required for this study in accordance with the national legislation and the institutional requirements.

## Author Contributions

MT and MO designed the study. PC, IH, AE, SB, MT, and MO performed the study, collected, analyzed and interpreted the data. PC and MO drafted the manuscript. All authors critically revised the manuscript. All authors contributed to the article and approved the submitted version.

## Funding

Funded by personal and departmental funds MO.

## Conflict of Interest

MT reports receiving a grant from the Swiss National Science Foundation, and having research collaborations with Roche, Novartis, and Idorsia (all outside of the submitted work). MO reports receiving grants from the Swiss National Science foundation outside of the submitted work, and consulting fees from Pharming Technologies B.V. during the conduct of the study and grants from Pharming Biotechnologies B.V. outside the submitted work. AE reports receiving grants from the Swiss National Science foundation outside of the submitted work. No conflict of interest for this work.

The remaining authors declare that the research was conducted in the absence of any commercial or financial relationships that could be construed as a potential conflict of interest.

## Publisher’s Note

All claims expressed in this article are solely those of the authors and do not necessarily represent those of their affiliated organizations, or those of the publisher, the editors and the reviewers. Any product that may be evaluated in this article, or claim that may be made by its manufacturer, is not guaranteed or endorsed by the publisher.

## Supplementary Material

Supplementary Figure 1 Receiver-operator characteristics (ROC) analysis of alternative pathway activity according to the composite outcome of mechanical ventilation and/or in-hospital death.
