# Supplementary figures and images for: Functional Activity of the Complement System in Hospitalized COVID-19 Patients: A Prospective Cohort Study
Source: Front Immunol. 2021 Oct 28;12:765330. doi: 10.3389/fimmu.2021.765330 (PMC8581394; doi:10.3389/fimmu.2021.765330)

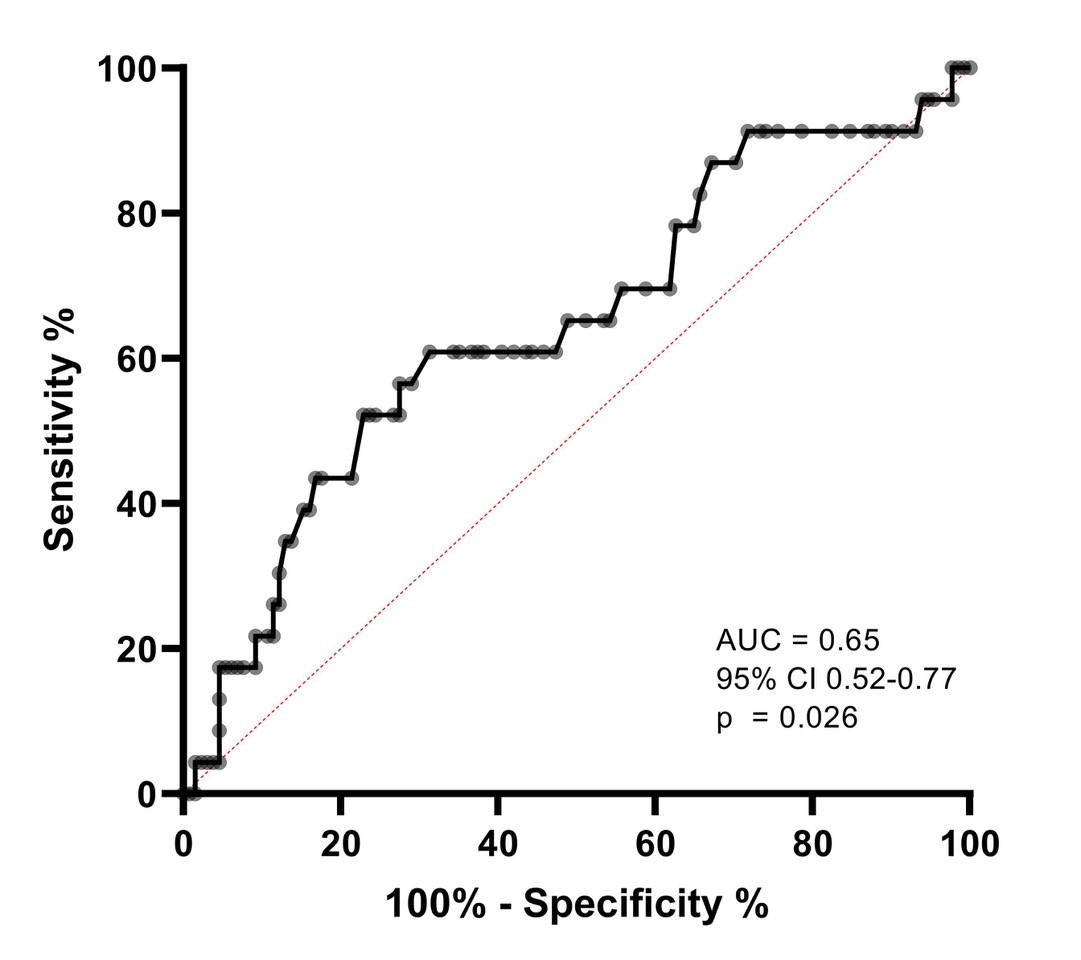

Supplement: Supplementary Figure 1 — Receiver-operator characteristics (ROC) analysis of alternative pathway activity according to the composite outcome of mechanical ventilation and/or in-hospital death. [file Image_1.jpeg]
